# Open management of neglected inferior dislocation of the shoulder with proximal humeral fracture in an adolescent

**DOI:** 10.1007/s11751-012-0151-6

**Published:** 2013-01-11

**Authors:** Shabir Ahmed Dhar, Sharief Ahmed Wani, Tahir Ahmed Dar, Shahid Hussain, Reyaz Ahmed Dar, Abdul Rouf Malik

**Affiliations:** 1 The Government Hospital of Bone and Joint Surgery, Srinagar, India; 2 The Skims MC, Bemina, Srinagar, India; 3 Department of Orthopaedics, Skims MC, Bemina, Srinagar, Kashmir India

**Keywords:** Fracture, Inferior dislocation, Bone shortening

## Abstract

Neglected dislocation of the shoulder is a rare condition with some cases of anterior and posterior dislocation being reported. We report a case with a fracture dislocation of the proximal humerus with the dislocated head lying inferior to the glenoid. We also report on the surgical management of a case with this extremely rare condition which required shortening of the distal fragment to reduce tissue tension.

## Introduction

Neglected shoulder dislocation is a rare condition [1]. Neglected dislocation of the shoulder when reported in literature is usually of the anterior type with very few posterior types reported [2, 3].

A pertinent fact that usually causes neglect is the fact that the patient is usually injured in high velocity accidents and presents with more obvious accompanying injuries [4, 5].

Literature points to the fact that these injuries are difficult to manage. The exact method of management is a matter of debate and discussion.

We report a case of fracture dislocation of the shoulder where the joint itself had undergone inferior dislocation. This case was operatively managed 4 weeks after the trauma and required bone shortening to reduce tissue tension.

## Case report

**Fig. 1:**
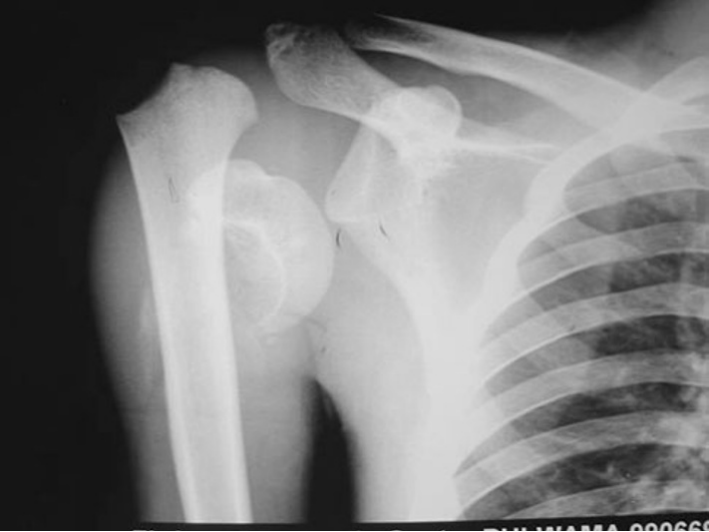
Radiograph of the affected shoulder at 4 weeks

**Fig. 2:**
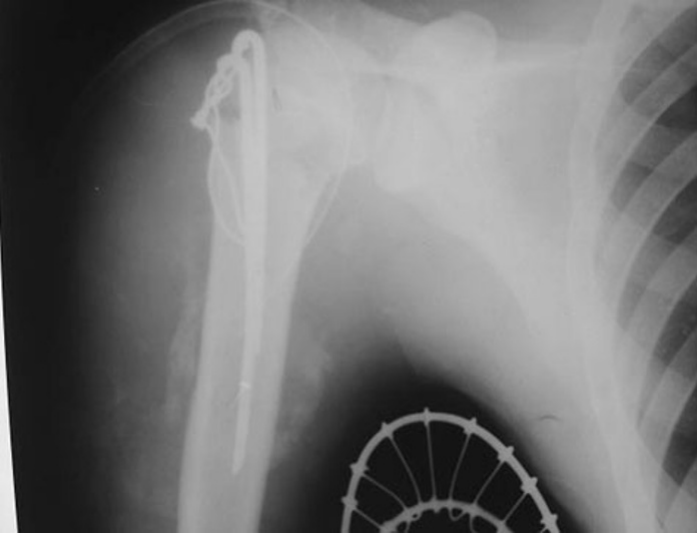
Post operative radiograph showing acceptable reduction

**Fig. 3:**
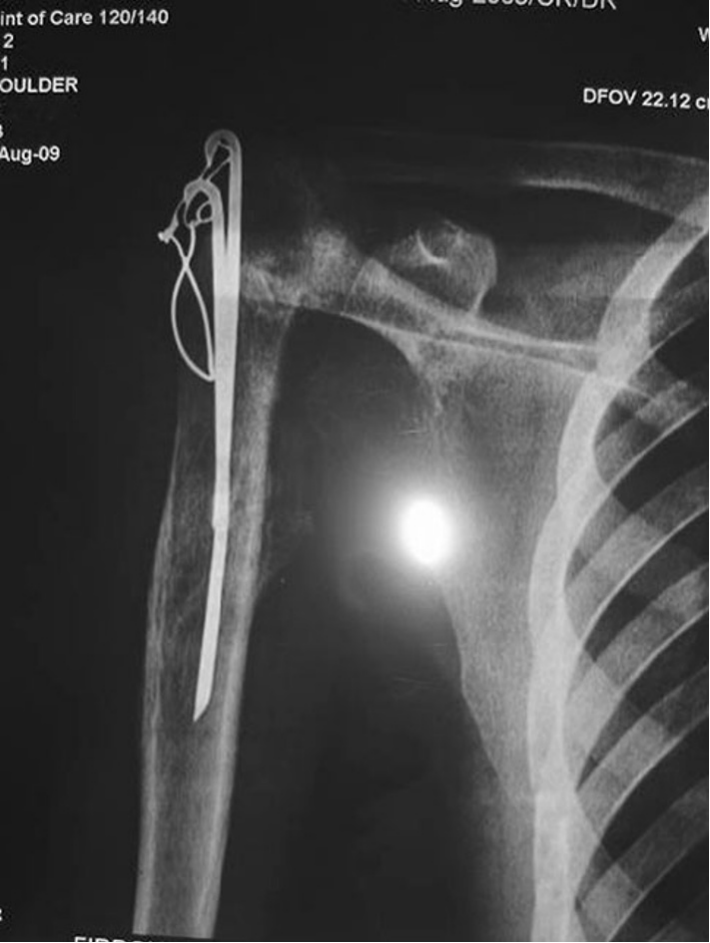
Radiograph 1 year after surgery

An 18-year-old male reported to our emergency department with a history of pain in the right shoulder and inability to move it without significant discomfort. He reported that he had been assaulted 4 weeks before the presentation and had sustained head injuries. He had been admitted elsewhere for the management of those injuries and had been managed on an indoor basis for 16 days. His shoulder discomfort had not been thought to be serious during his stay in that hospital. Orthopaedic consultation had been recommended at the time of discharge. Examination revealed a reasonably maintained contour of the shoulder with total restriction of abduction. The patient was able to internally rotate the shoulder up to 20° and external rotation was not possible (Figs. 1, 2, 3).

Radiographs of the shoulder showed that the proximal humerus was fractured, and the proximal end of the distal fragment had migrated proximally to lie under the deltoid giving it a near normal contour. The head was dislocated inferiorly. This was confirmed by the axillary view. A significant callus had formed around the head and the shaft at their new position of contact.

It was obvious that the proximal pull on the distal fragment had made it migrate proximally.

According to these findings, open reduction and reconstruction of the proximal humerus were considered necessary. Under general anaesthesia, the patient was placed in a supine position and the glenohumeral joint was assessed via a deltopectoral approach. The axillary nerve was palpated to ascertain its position. The long head of the biceps was still intact. The glenoid cavity and the proximal humerus were exposed, and granulation tissue and the callus were cleared from it. After meticulous removal of the scar tissue, the glenoid articular cartilage looked to be in good condition and the humeral head was reduced.

As expected, it was not possible to reduce the distal fragment to its normal position beneath the head of the humerus. The end was stripped of soft tissue and shortened by 2 cm. This made the reduction possible beneath the head. The supraspinatus was reattached to the tuberosity area. The other muscles were reefed along with the capsule of the shoulder joint.

The wound was covered in layers, and the shoulder immobilised in 45° of internal rotation. After 4 weeks, pendular exercises were started with gradual institution of range of motion exercises. The final range of motion was recorded after 1 year when the patient had a more than normal internal rotation with an external rotation of 15°. However, abduction was only 25° at the joint. The flexion was 70° and the extension 20°. The patient was pain free and was able to perform his routine duties.

The X-ray, however, showed a gradual superior subluxation of the shoulder joint which was suggestive of undue tissue tension and rotator cuff insufficiency.

## Discussion

Shoulder dislocation is an injury which is at risk of being neglected in a patient with more severe injuries. The potential of missed diagnosis is always there [5].

Looking at literature, the neglect of this dislocation is thankfully rarely reported. There is, however, significant variability of this injury. Shoulder hemiarthroplasty has been used with these injuries [6]. However, it is recommended that the head should be preserved in young patients [5]. Sometimes neglect of these injuries is recommended with acceptance of subnormal function [7].

Inferior glenohumeral dislocation is unusual accounting for approximately 0.5 % of all shoulder dislocations. Tomovic et al. [8] in a series of 248 shoulder dislocations found 244 anterior dislocations (98.4 %), three posterior dislocations (1.2 %), and 1 inferior glenohumeral dislocation associated with a fracture of the greater tuberosity of the humerus (0.4 %).

Musculoskeletal injuries associated with luxatio erecta are commonly reported in the literature and may include avulsion of the shoulder capsule; disruption of the supraspinatus, infraspinatus, subscapularis, teres minor, and pectoralis major; fractures of the clavicle, body of the scapula, coracoid, acromion, inferior glenoid, humeral head, and greater tuberosity of the humerus; and acromioclavicular dislocation.

Radiographic evaluation reveals a glenohumeral dislocation. The position of the humeral head is variable. It may lie at or beneath the inferior rim of the glenoid. Further displacement to the level of the surgical neck of the scapula and against the rib cage at the level of the third or fourth intercostal space may also be seen [8].

A fresh inferior dislocation can be reduced by traction in the long axis of the arm with pressure applied in the axilla simultaneously [9]. However, due to the rarity of this condition, many orthopaedicians are unfamiliar with this technique [10].

Surgical management is difficult in view of the fact that chronicity causes significant attenuation of the structures around the shoulder joint. Our case is the first report, in our opinion, where the head was dislocated inferiorly, and the injury had been neglected for 4 weeks. Even though we were able to lever the humeral head reasonably easily into the glenoid cavity, but reduction of the proximally migrated fragment required shortening of this bone to reduce tissue tension. Balancing this tissue tension without any clearly defined criteria is empirical. The guideline that we followed on the operating table was the ability to move the joint through a passive range of motion without any subluxation. We concur with the previous reports in that the injury is difficult to manage and often occurs due to imperfect examination of the poly-trauma patient at the outset.
